# Barriers to healthcare utilization in fatiguing illness: a population-based study in Georgia

**DOI:** 10.1186/1472-6963-9-13

**Published:** 2009-01-20

**Authors:** Jin-Mann S Lin, Dana J Brimmer, Roumiana S Boneva, James F Jones, William C Reeves

**Affiliations:** 1 Chronic Viral Diseases Branch, National Center for Zoonotic, Vector-borne and Enteric Diseases, Centers for Disease Control and Prevention, Mail Stop A-15, 1600 Clifton Rd. NE, Atlanta, GA 30333, USA

## Abstract

**Background:**

The purpose of this study was to determine the prevalence of barriers to healthcare utilization in persons with fatiguing illness and describe its association with socio-demographics, the number of health conditions, and frequency of healthcare utilization. Furthermore, we sought to identify what types of barriers interfered with healthcare utilization and why they occurred.

**Methods:**

In a cross-sectional population-based survey, 780 subjects, 112 of them with chronic fatigue syndrome (CFS), completed a healthcare utilization questionnaire. Text analysis was used to create the emerging themes from verbatim responses regarding barriers to healthcare utilization. Multiple logistic regression was performed to examine the association between barriers to healthcare utilization and other factors.

**Results:**

Forty percent of subjects reported at least one barrier to healthcare utilization. Of 112 subjects with CFS, 55% reported at least one barrier to healthcare utilization. Fatiguing status, reported duration of fatigue, insurance, and BMI were significant risk factors for barriers to healthcare utilization. After adjusting for socio-demographics, medication use, the number of health problems, and frequency of healthcare utilization, fatiguing status remained significantly associated with barriers to healthcare utilization. Subjects with CFS were nearly 4 times more likely to forego needed healthcare during the preceding year than non-fatigued subjects while those with insufficient fatigue (ISF) were nearly 3 times more likely.

Three domains emerged from text analysis on barriers to healthcare utilization: 1) accessibility; 2) knowledge-attitudes-beliefs (KABs); and, 3) healthcare system. CFS and reported duration of fatigue were significantly associated with each of these domains. Persons with CFS reported high levels of healthcare utilization barriers for each domain: accessibility (34%), healthcare system (25%), and KABs (19%). In further examination of barrier domains to healthcare utilization, compared to non-fatigued persons adjusted ORs for CFS having "accessibility", "KAB" and "Healthcare System" barrier domains decreased by 40%, 30%, and 19%, respectively.

**Conclusion:**

Barriers to healthcare utilization pose a significant problem in persons with fatiguing illnesses. Study results suggested two-fold implications: a symptom-targeted model focusing on symptoms associated with fatigue; and an interactive model requiring efforts from patients and providers to improve interactions between them by reducing barriers in accessibility, KABs, and healthcare system.

## Background

Chronic fatigue syndrome (CFS) comprises a complex problem for health care providers and patients. The illness is defined based on symptoms and ruling-out medical and psychiatric conditions with similar clinical characteristics [1,2]. There are no characteristic clinical signs or diagnostic laboratory abnormalities, the pathophysiology remains inchoate, and management focuses on treating symptoms and rehabilitation [3]. Approximately 2.5% of adults in the United States may suffer from CFS and almost 4% report symptoms compatible with CFS [2,4]. Most people with CFS identified in population-based studies have been ill for 5 to 7 years. They are profoundly functionally impaired; 25% are unemployed or receiving disability because of their illness, and families in which a member suffers CFS forego $20,000 in annual earnings and wages [4-7]. In spite of the burden imposed by CFS on individuals and the population, less than 20% of those with the illness have been diagnosed and received treatment [6,8].

Barriers to healthcare utilization are particularly important. Minorities, the uninsured, and persons living in rural areas are at greater risk of not seeking needed healthcare. For example, blacks and Hispanics are less likely to seek medical care for depression than whites, although healthcare providers recommend treatment at equal rates [9]. Compared to whites, blacks are less likely to have health insurance and they also may perceive their access to healthcare differently [10,11]. Individuals may delay seeking healthcare due to cultural differences, knowledge concerning their illness, or perceptions of the healthcare system. Barriers to influenza vaccination for blacks included issues of mistrust whereas for Hispanics, barriers focused on access and cost [12].

There are at least three reasons as to why people with CFS have not been diagnosed: 1) barriers within the medical community (e.g., lack of knowledge concerning diagnosis and treatment); 2) lack of access to healthcare (e.g., lack of health insurance); and 3) barriers to healthcare utilization (e.g., lack of time, long working hours) [13]. In a Chicago community-based study with a sample of 32 CFS patients, over one-third of subjects with CFS never consulted a physician for their fatigue [14]. Reasons for not seeking medical care were limited social and economic resources, lack of knowledge of CFS among physicians, inappropriate diagnoses, and a feeling that minimal benefit is gained from seeking traditional healthcare [14]. In a sample of 47 adults with CFS recruited from several sources, 81% of CFS subjects reported at least one barrier to service access [15]. Six barriers are identified in this study: lack of financial/insurance resources, lack of knowledge of service availability, travel distance and lack of transportation, problems with service providers, and CFS-associated impairment [15]. While both of these studies document the importance of barriers to healthcare utilization in CFS, small sample sizes prevent generalizability.

There has been limited effort to estimate the occurrence of barriers to healthcare utilization among persons with fatiguing illness including CFS. This article addresses these needs by evaluating: 1) the prevalence of barriers to healthcare utilization in persons with fatiguing illness identified from a large stratified random population sample in the state of Georgia; 2) nature (domains) of the barriers; and 3) associations between these barriers and fatiguing status, socio-demographic, the number of health conditions, and frequency of healthcare utilization. This knowledge is necessary to devise strategies for reducing barriers to healthcare utilization.

## Methods

The study adhered to the human experimental guidelines of the US Department of Health and Human Services and the Helsinki Declaration. The Human Subjects Committee of the Centers for Disease Control and Prevention approved the study protocol, and all subjects gave informed consent.

### Study Design and Sample

The data derive from a large cross-sectional population-based study of CFS and chronic unwellness in Georgia, investigating the prevalence of CFS between September 2004 and July 2005 [2]. Although the source study is an investigation of the prevalence of CFS, the present study uses the healthcare utilization data from the source study to investigate the prevalence of barriers to healthcare utilization. The details of the source study have been published [4] but are summarized here.

In Phase 1, a random sample of 10,837 households (with 21,165 members in which at least one household member was aged 18 to 59) was included in a cross-sectional, screening survey that utilized Computer Assisted Telephone Interviews (CATI). Based on the household screening survey, 8,862 adults were selected for detailed telephone interviews; 5,630 individuals completed the detailed telephone interview; 1874 refused to participate, 134 were ineligible, and 1272 were excluded due to physical/mental impairment, unable to contact, language barriers, and deceased – an overall response rate of 75%.

Phase 2 comprised a one-day clinical assessment that included a standard medical and psychiatric evaluation and healthcare utilization questionnaire [1,2,4]. Based on the detailed telephone interviews from Phase 1, study subjects entering the clinical study phase had been classified as: 1) CFS-like, 2) Chronically Unwell, and 3) Well [4]. All 469 persons with a CFS-like illness were invited for clinical evaluation and 292 (62%) participated.

Two hundred sixty-eight randomly selected chronically unwell telephone interview respondents completed the clinical evaluation. Finally, 223 individuals classified as well on telephone interview, who were matched to the CFS-like based on residence (metropolitan, urban, rural), sex, race/ethnicity and age (within 3 years) completed clinic evaluations. The matching of the source study was done for the investigation of the prevalence of CFS.

Based on their clinical evaluation, subjects were identified with medical comorbid conditions such as previously undiagnosed thyroid disease (24% of the total), anemia (18%), uncontrolled diabetes (14%), autoimmune disease (11%), inflammatory disease (8%), heart disease (7%), arthritis (3%) and pulmonary disease (3%); and psychiatric comorbid conditions encompassed previously undiagnosed alcohol or substance abuse (43%), melancholic depression (26%), bipolar disorder (19%), psychosis (7%), and anorexia/bulimia (5%). To examine the impact of co-existing health conditions with fatiguing illness on healthcare utilization, the fatiguing status of subjects were identified as: 1) CFS, 2) CFS, but for an exclusionary diagnosis (subjects who fulfill empiric criteria for CFS but who have an exclusionary diagnosis), 3) insufficient fatigue (ISF) (subjects who have been ill for > 6 months, but do not fulfill empiric criteria for CFS), 4) ISF with otherwise exclusionary conditions, or 5) non-fatigued (NF). CFS was diagnosed as specified in the 1994 international research case definition [1] using validated instruments as specified by the International CFS Study Group [2] and current CDC standards [2,4]. Exclusionary conditions could not be determined for two persons due to incomplete lab results. One person only partially completed the healthcare utilization survey and was excluded from the analysis, for a total of 780 respondents.

### Measures

#### Healthcare Utilization

We defined healthcare utilization by responses to the question "During the past year, did you see, talk to, or consult with a healthcare professional about your personal health?" and then "During the past year, how many times did you see, talk to, or consult with a healthcare professional about your personal health." Respondents were asked about frequencies of consulting with a healthcare professional because of problems with four CFS defining symptoms: fatigue, sleep, memory or concentration, and pain. Respondents who reported foregoing healthcare were asked to indicate reasons for seeking healthcare, and why they did not seek healthcare. These responses were recorded as open-ended text. Subjects selected for the clinical evaluation completed the Healthcare Utilization Questionnaire at home prior to their clinic visit. At the clinic, study coordinator reviewed the responses to assure the understanding of questions and logic of skip pattern and worked with subjects to rectify omissions and errors if necessary.

#### Barriers to Healthcare Utilization

The primary outcome was a binary indicator of the occurrence of barriers to healthcare utilization. If a respondent reported foregoing healthcare (i.e., wanted to see a healthcare professional but did not), then he or she was identified as having barriers to healthcare utilization. The secondary outcome variables included three barrier domains to healthcare utilization that emerged as theme categories from the text (verbatim) responses in open-ended questions regarding reasons for not seeking needed healthcare. If a respondent reported having at least one barrier type in a domain, then the presence of a barrier was established. An aggregated variable for the count of the number of barrier types was also created and ranged from 0 (no barrier) to 11 (maximum).

#### Socio-demographics, Health History and Health Status

We identified the following as correlates to barriers to healthcare utilization: 1) Socio-demographic characteristics – sex, age, race, residential areas (metropolitan, urban, and rural), marital status, parental status (children under 18 vs. no children under 18), work status, income, education, and insurance; 2) Health history – history of CFS diagnosis (a self-reported diagnosis derived from the question, "Have you ever been diagnosed with chronic fatigue syndrome?"), reported duration of fatigue, current CFS diagnosis (met 1994 CFS case definition at the clinic visit), use of over-the-counter (OTC) pain relievers; and 3) Health status – Body Mass Index (BMI), Physical Component Summary (PCS) and Mental Component Summary (MCS) scores, and the number of health problems (medical or psychiatric conditions). PCS and MCS scores are indicators of health status with PCS measuring wellness or illness and MCS for mental health status (i.e., depression). The PCS and MCS scores were derived from the 8 dimensions of the Medical Outcomes Survey Short Form (SF-36). PCS and MCS are a linear combination of 8 SF-36 scales; PCS is predominantly based on the scales Physical Functioning, Role Physical, Bodily Pain and General Health Perceptions while MCS is predominantly based on the scales Mental Health, Role Emotional, Social Functioning and Vitality) (range 0–100, 100 = optimal) [16].

In this study, fatiguing status is the exposure variable of interest and confounding variables include socio-demographics, the number of health conditions, and frequency of healthcare utilization.

### Statistical Analysis

#### Qualitative Analysis

We analyzed all text (verbatim) responses with SPSS Text Analysis for surveys 2.0 (Chicago, IL: SPSS Inc, 2005) [17]. In SPSS module for text analysis, the emerging categories were extracted by combined methods: 1) a semantic network approach based on Wordnet; and 2) "term inclusion" that creates "categories using lexical series algorithms" (Chicago, IL: SPSS Inc, 2005). After automatic extractions, manual review was done for each category to reduce the misclassification of theme categories automatically extracted via the software through term, pattern, and contextual qualifier. The manual review was conducted by a statistician (Dr. Lin) and a CFS research clinician (Dr. Jones). If a potential misclassification was observed by the first manual reviewer, the second reviewer would consolidate the discrepancies with the first reviewer. Finally, the categories of text responses were exported as dichotomous variables into Microsoft Excel format and then imported to SAS Version 9.1 for subsequent data analyses (SAS Institute Inc, Cary, NC).

#### Quantitative Analysis

An exact 95% confidence interval for a binomial proportion was reported for the estimated prevalence of barriers to healthcare utilization. Univariate logistic regression was performed to examine the bivariate association between barrier status and factors such as fatiguing status, socio-demographic characteristics, the number of health conditions, and frequency of healthcare utilization. Multivariate analyses were conducted using separate logistic regression models for each outcome: the presence of barriers to healthcare utilization, and the presences of three domains, with fatiguing status as the main independent variable adjusted for covariates. All tests of significance were two-tailed with the alpha level set at 0.05.

## Results

The median age of 780 subjects was 45 years (mean 44, SD 10, range 18–59). The majority were women (76%), white (71%), and racial diversity of the sample reflects the Georgia population [18] (Table 1). Sixty-four percent of subjects were married and about half reported no children under the age of 18. The study sample was well-educated with 94% having at least a high school education. Over 70% of subjects were employed either full- or part-time, and 57% of subjects met or surpassed the Georgia median household income ($42,679). Eighty-five percent reported being insured and of the insured 5% had multiple types of healthcare coverage.

**Table 1:** Socio-demographics Characteristics and Barriers to Healthcare Utilization

Characteristics		**Barriers to Healthcare Utilizations**			
		
	**All**	**Yes**	**No**	**Unadjusted Odds Ratios^a ^** **(95% CI)**	**Adjusted Odds Ratios^b ^** **(95% CI)**
	**n = 780**	**n = 315 (40%)**	**n = 465 (60%)**		
Age, No. (%)			Row %	Row %	
18–29	93 (11.92)	45.16	54.84	1.38 (0.85–2.22)	1.32 (0.77–2.26)
30–39	147 (18.85)	41.50	58.50	1.19 (0.78–1.79)	0.94 (0.59–1.50)
40–49	281 (36.03)	40.93	59.07	1.16 (0.82–1.64)	1.02 (0.69–1.46)
50–59	259 (33.20)	37.45	62.55	Reference	Reference
Sex					
Female	594 (76.15)	42.09	57.91	1.35 (0.96–1.91)	**1.47 (1.02–2.12)**
Male	186 (23.85)	34.95	65.05	Reference	Reference
Race					
Black	194 (24.87)	39.18	60.82	0.94 (0.67–1.31)	0.93 (0.64–1.37)
Other	35 (4.49)	42.86	57.14	1.10 (0.55–2.18)	1.04 (0.50–2.15)
White	551 (70.64)	40.65	59.35	Reference	Reference
Residential Areas					
Rural	383 (49.10)	41.78	58.22	1.42 (0.94–2.15)	1.49 (0.94–2.35)
Urban	266 (34.10)	41.73	58.27	1.42 (0.92–2.19)	1.48 (0.93–2.35)
Metropolitan	131 (16.79)	33.59	66.41	Reference	Reference
Marital Status					
Married	502 (64.36)	39.44	60.56	1.12 (0.83–1.50)	1.13 (0.80–1.60)
Not Currently Married	278 (35.64)	42.09	57.91	Reference	Reference
Parental Status					
Children under 18	400 (51.28)	42.25	57.75	1.17 (0.88–1.56)	1.24 (0.89–1.72)
No Children under 18	380 (48.72)	38.42	61.58	Reference	Reference
Work Status					
Full-time	487 (62.44)	40.25	59.75	1.00 (0.72–1.38)	1.20 (0.83–1.72)
Part-time	80 (10.26)	41.25	58.75	1.04 (0.62–1.75)	0.96 (0.55–1.67)
Not currently working	213 (27.31)	40.38	59.62	Reference	Reference
Education					
Less than High School	46 (5.90)	41.30	58.70	1.04 (0.57–1.91)	0.90 (0.47–1.73)
High School or Above	734 (94.10)	40.33	59.67	Reference	Reference
Household Income					
Below GA Median Income^c^	323 (43.01)	41.49	58.51	1.10 (0.82–1.47)	0.92 (0.65–1.30)
GA Median Income or Above	428 (56.99)	39.25	60.75	Reference	Reference
Insurance*					
Uninsured*	**113 (14.51)**	**53.10**	**46.90**	**1.83 (1.22–2.73)**	**2.03 (1.28–3.21)**
Insured	666 (85.49)	38.29	61.71	Reference	Reference
Multiple Healthcare Coverage*					**Not included**
Two or more*	**38 (4.88)**	**28.95**	**71.05**	**0.36 (0.16–0.80)**	**--**
One*	**628 (80.62)**	**38.85**	**61.15**	**0.56 (0.38–0.84)**	**--**
None	113 (14.51)	53.10	46.90	Reference	**--**

*indicates p-value < 0.01 (two-tailed tests); p-value < 0.05 were in bold font.

*Note*: Values are No. (%) unless otherwise indicated.

^a ^Comparison between subjects who having any barrier to healthcare and those who not.

^b ^Adjusted for all other variables in the table.

^c ^Georgia median income is $42,679.

### Domains of Barriers to Healthcare Utilization

In the text analysis, nine themes emerged from reasons for barriers to healthcare utilization and these themes were classified into three domains: accessibility, knowledge-attitudes-beliefs (KABs), and healthcare system.

#### Accessibility

The accessibility domain includes physical constraints such as family and work responsibilities that interfered with seeking help, geographical location (not enough providers in an area), difficulty obtaining transportation to the provider's office, difficulty obtaining a timely appointment to see a provider, and inconvenient office hours. This domain also included financial concerns about cost, insurance company co-payment, and that insurance would not cover the care received.

#### Knowledge, Attitudes, and Beliefs (KABs)

The primary knowledge barrier consisted of both those with the illness and healthcare professionals overlooking a fatiguing illness due to lack of knowledge about such illnesses. Attitudinal barriers included subjects' thinking that the problem was "no big deal" or would "get better on its own," and that individuals needed an excuse or a better reason to see a healthcare professional. Personal barriers (minimization of illness and lack of family support) and fear (fear of stigmatization and confronting the problem) were additional beliefs held by study subjects.

#### Healthcare System

The third domain, healthcare systems, comprised themes of trust and confidence, structural or system barriers, and self-diagnosis/self-treatment. Subjects indicated that trust and confidence in healthcare professionals impacted their decision not to seek healthcare consultation: a doctor may not do enough to find out what is making them sick; the treatment did not make them feel better; a healthcare professional will require more tests without reviewing previous test results; belief that doctors did not believe in the diagnosis of CFS; and subjects felt dejected by healthcare professionals or that healthcare professionals might minimize their illness.

Structural/system barriers in this category included lack of referral system and insensitivity to patient needs. In terms of self-diagnosis and self-treatment barriers, subjects sometimes self-diagnosed their symptoms or illness and considered them as consequences of lack of exercise, overweight, aging, hormone imbalance, depression, pre-menopause, menopause and intermittent pain. As a consequence of foregoing healthcare and self-diagnosis, subjects self-treated themselves. One of the commonly used self-treatments of their symptoms or illness was using over-the-counter medications to treat their "comes and goes" pain.

The top seven themes among all barrier domains consisted of attitudes (9.7%), self-diagnosis (9.6%), finance (9.5%), time constraint (8.3%), healthcare coverage (7.3%), fear (5.9%), and lack of trust and confidence in their health professionals (5%).

### Unadjusted Percentages of Having Any Barrier and Barrier Domains

Overall, 40% of subjects reported at least one barrier to healthcare utilization (Table 2). Of 112 subjects with CFS, 55% reported at least one barrier to healthcare utilization. Significantly higher rates of perceived barriers to healthcare utilization occurred in the groups of "CFS but for an exclusionary diagnosis" (52%), ISF, and ISF with other exclusionary conditions (43% each). Among barrier domains, accessibility was the most common domain (22%), followed by the healthcare system domain (16%), and the KAB domain (15%). A quarter of respondents reported barriers from one domain, 10% of respondents reported two, and 2.3% of respondents reported barriers from all three domains. Among subjects with CFS, accessibility domain (34%) had the highest percentage of barriers to healthcare utilization, followed by the healthcare system domain (25%), and the KAB domain (19%).

**Table 2:** The Unadjusted Percentages of Each Domain of Barriers to Healthcare Utilization

Barrier Domain	Accessibility^b^		KAB		Healthcare System		Any Barrier	
	
	P	95% CI^a ^for p	p	95% CI for p	p	95% CI for p	p	95% CI for p
CFS (n = 112)	38.4%	(29.4%–48.1%)	19.6%	(12.7%–28.2%)	26.8%	(18.9%–36.0%)	55.4%	(45.7%–64.8%)
CFS but for an exclusionary DX (n = 100)	30.0%	(21.2%–40.0%)	19.0%	(11.8%–28.1%)	22.0%	(14.3%–31.4%)	52.0%	(41.8%–62.1%)
ISF with Exclusionary Conditions (n = 157)	22.9%	(16.6%–30.3%)	14.7%	(9.5%–21.2%)	14.7%	(9.5%–21.2%)	43.3%	(35.4%–51.5%)
ISF (n = 264)	20.1%	(15.4%–25.4%)	16.3%	(12.1%–21.3%)	15.9%	(11.7%–20.9%)	42.8%	(36.8%–49.0%)
Non-fatigued (n = 147)	7.5%	(3.8%–13.0%)	4.8%	(1.9%–9.6%)	4.8%	(1.9%–9.6%)	13.6%	(8.5%–20.2%)
Total (n = 780)	22.2%	(19.3%–25.3%)	14.6%	(12.2%–17.3%)	15.9%	(13.4%–18.7%)	40.4%	(36.9%–43.9%)

^a ^95% exact binomial confidence interval (CI).

^b ^If a respondent reported at least one type of barriers in the Accessibility domain, he/she was identified as having barrier in that domain; otherwise none.

### Socio-demographics and Barriers to Healthcare Utilization

Table 1 also presents unadjusted odds ratios and adjusted odds ratios for all other variables in the table. There were no statistically significant differences in reported barriers to healthcare with respect to age, race, residential areas, marital status, parental status, work status, income, and education. Women were more likely than men to report not consulting a healthcare professional as needed (adjusted OR = 1.47, 95% CI = 1.02–2.12). Compared to insured subjects, those without insurance were more likely to forego healthcare (adjusted OR = 2.03, 95% CI = 1.28–3.21).

### Health History and Status and Barriers to Healthcare Utilization

Table 3 shows unadjusted odds ratios and adjusted odds ratio for all other variables in the table. One hundred twelve (14%) subjects were classified as CFS, based on clinical evaluation, yet only 16 (14%) of those with CFS reported having been diagnosed as CFS by a physician. Interestingly, 21% of 100 subjects who were classified as "CFS but for an exclusionary diagnosis" reported having been diagnosed as CFS by a physician.

**Table 3:** Health History and Status of Study Population (n = 780), and Their Bivariate Association with Barriers to Healthcare Utilization

**Characteristics**		**Barriers to Healthcare Utilization**			
		
	**All**	**Yes**	**No**	**Unadjusted Odds Ratios^a^**	**Adjusted Odds Ratios^b^**
	**n = 780**	**n = 315 (40%)**	**n = 465 (60%)**	**(95% CI)**	**(95% CI)**
Fatiguing Status***		**Row %**	**Row %**		
CFS**	**112 (14.36)**	**55.36**	**44.64**	**7.87 (4.32–14.35)**	**4.88 (2.42–9.86)**
CFS but an exclusionary DX*	**100 (12.82)**	**52.00**	**48.00**	**6.88 (3.72–12.70)**	**5.15 (2.48–10.69)**
**ISF with Exclusionary Conditions**	**157 (20.13)**	**43.31**	**56.69**	**4.85 (2.75– 8.55)**	**3.97 (2.14–7.36)**
**ISF**	**264 (33.85)**	**42.80**	**57.20**	**4.75 (2.79– 8.08)**	**3.81 (2.17–6.72)**
**Non-fatigued**	147 (18.85)	13.61	86.39	Reference	Reference
Reported Duration of Fatigue***					
>= 6 months*	**396 (50.77)**	**52.02**	**47.98**	**2.85 (2.11–3.86)**	**1.82 (1.25–2.65)**
**<6 months**	21 (2.69)	42.86	57.14	1.97 (0.81–4.83)	1.65 (0.65–4.18)
Never	363 (46.54)	27.55	72.45	Reference	Reference
Other Health Conditions					**Not included**
Both Psychiatric and Medical	33 (4.23)	54.55	45.45	1.89 (0.93–3.84)	**--**
Psychiatric	114 (14.62)	48.25	51.75	1.47 (0.98–2.21)	**--**
Medical	133 (17.05)	36.09	63.91	0.89 (0.60–1.33)	**--**
None	500 (64.10)	38.80	61.20	Reference	**--**
BMI^c^*					
Obese (>= 29.9)	278 (35.64)	37.77	62.30	1.05 (0.74–1.51)	0.84 (0.57–1.25)
Overweight (24.9–29.9)*	**264 (33.85)**	**46.59**	**53.41**	**1.51 (1.06–2.17)**	1.31 (0.89–1.92)
Under/Normal Weight (<24.9)	238 (30.51)	36.55	63.45	Reference	Reference
History of CFS DX					
Yes	50 (6.44)	44.00	56.00	1.17 (0.66–2.09)	0.70 (0.37–1.34)
No	727 (93.56)	40.17	59.83	Reference	Reference
OTC^d ^Pain Relieves					
Taking	294 (37.69)	41.16	58.84	1.05 (0.78–1.41)	1.01 (0.74–1.39)
Not-taking	486 (62.31)	39.92	60.08	Reference	Reference
	Mean (SD)	Mean (SD)	Mean (SD)	Per Increment	
**Number of Health Problems**	**1.85 1.97**	**2.19 (2.14)**	**1.61 (1.81)**	**1.16 (1.08–1.25)**	1.09 (1.00–1.19)
Healthcare Utilization				Per Consultation	
Frequency of Consultation				Increment	
Consultation on Fatigue	1.38 (3.31)	1.60 (3.88)	1.22 (2.84)	1.04 (0.99–1.08)	1.01 (0.95–1.07)
Consultation on Sleep	0.94 (2.20)	1.03 (2.20)	0.88 (2.20)	1.03 (0.97–1.10)	0.99 (0.90–1.09)
Consultation on Cognition	0.59 (2.09)	0.57 (1.79)	0.60 (2.27)	0.99 (0.93–1.06)	0.93 (0.85–1.03)
Consultation on Pain	3.52 (7.92)	3.66 (8.74)	3.42 (7.32)	1.00 (0.99–1.02)	0.99 (0.96–1.01)

* indicates p-value < 0.01. **indicates p-value < 0.001. ***indicates p-value < 0.0001 (two-tailed tests); p-value < 0.05 were in bold font.

*Note*: Values are No. (%) unless otherwise indicated.

^a ^Comparison between subjects who having any barrier to healthcare and those who not.

^b ^Adjusted for all other variables in the table.

^c ^Body mass index (BMI) was calculated as weight in kilograms divided by height in meters squared.

^d ^OTC pain relieves included the over-the-counter (OTC) used for the reason of reducing pain such as joint, muscle, headache, backache, stomach ache, abdominal ache, arm and leg pain in the preceding two weeks.

Subjects with CFS were nearly 4 times more likely to forego needed healthcare during the preceding year than non-fatigued persons (adjusted OR = 4.88, 95% CI = 2.42–9.86, p < 0.001) while subjects with ISF were nearly 3 times more likely (adjusted OR = 3.81, 95% CI = 2.17–6.72, p < 0.01). Subjects with fatigue lasting 6 months or longer were almost twice as likely as those who never had fatigue symptom before to forego needed healthcare (adjusted OR = 1.82, 95% CI = 1.25–2.65, p < 0.01). Of the 112 participants classified as CFS, the sixteen who reported having been diagnosed as CFS by a physician had worse physical health outcome and better mental health outcome than those without a prior formal CFS diagnosis (PCS: 31.39 vs. 38.97 for history of CFS diagnosis or not, p-value = 0.0035; MCS: 44.67 vs. 37.25, p-value = 0.0282).

In general, overweight subjects were about 30% more likely to forego needed healthcare than under/normal weight subjects (unadjusted OR = 1.51, 95% CI = 1.06–2.17; adjusted OR = 1.31, 95% CI = 0.89–1.92). When considering number of health problems, every health problem increases 9% of the likelihood of forgoing needed healthcare (unadjusted OR = 1.16, 95% CI = 1.08–1.25; adjusted OR = 1.09, 95% CI = 1.00–1.19). We did not find any significant association between barriers and frequency of healthcare utilization in fatigue, sleep, cognition, and pain.

### Fatiguing Status and Barrier Domains to Healthcare Utilization

Table 4 summarizes the results of multivariate models for the likelihood of having at lease one barriers, and three barrier domains to healthcare utilization by fatiguing status compared to non-fatigued persons. Persons with CFS were more likely than non-fatigued persons to report barriers in each domain to healthcare utilization. After adjusting for socio-demographics, medication use, the number of health problems, and frequency of healthcare utilization, the adjusted odds ratios differed compared to the unadjusted odds ratios with the exception of KAB barrier domain. Persons with CFS were 4 times more likely than non-fatigued persons to report barriers to healthcare utilization. In further examination of the types of barriers to healthcare utilization, the adjusted odds ratios for the presence of the "accessibility" barrier domain significantly decreased 42% from the unadjusted ORs for the CFS classified subjects compared to the non-fatigued group (unadjusted OR = 7.71 and adjusted OR = 4.59). Compared to the non-fatigued group, adjusted OR for having barriers in the "KAB" and "Healthcare System" were respectively decreased 30% and 19% for subjects with CFS.

**Table 4:** Unadjusted Odds Ratio and Adjusted Odds Ratio for Barriers to Healthcare Utilization by Fatiguing Status

Fatiguing status	Unadjusted Odds Ratio (95% CI)^a^			
	
	Any Barrier	Accessibility	KAB	Healthcare System
**CFS**	7.87 (4.32–14.35)**	7.71 (3.74–15.87)***	**4.89 (2.01–11.91)**	7.32 (3.08–17.41)**
CFS but an exclusionary DX	6.88 (3.72–12.70)*	**5.30 (2.51–11.20)**	**4.69 (1.89–11.64)**	**5.64 (2.31–13.80)**
ISF with Exclusionary	**4.85 (2.75– 8.55)**	**3.68 (1.79– 7.54)**	**3.43 (1.43– 8.26)**	**3.43 (1.43– 8.26)**
Conditions	**4.75 (2.79– 8.08)**	**3.11 (1.57– 6.16)**	**3.89 (1.70– 8.89)**	**3.78 (1.65– 8.66)**
ISF	Reference	Reference	Reference	Reference
Non-fatigued				

	Adjusted^b ^Odds Ratio (95% CI)			
	
	Any Barrier	Accessibility	KAB	Healthcare System

**CFS**	**5.38 (2.59–11.17)**	4.59 (1.86–11.32)*	**3.44 (1.19– 9.89)**	5.93 (2.16–16.27)*
CFS but an exclusionary DX	**5.41 (2.51– 11.66)**	**3.00 (1.16– 7.78)**	**5.88 (1.97–17.55)**	**4.94 (1.69–14.39)**
ISF with Exclusionary	**4.02 (2.11– 7.64)**	**2.89 (1.26– 6.61)**	**3.45 (1.32– 8.98)**	**2.78 (1.06– 7.25)**
Conditions	**3.72 (2.08– 6.66)**	2.03 (0.94– 4.38)	**3.44 (1.43– 8.30)**	**3.21 (1.34– 7.74)**
ISF	Reference	Reference	Reference	Reference
Non-fatigued				

*indicates p-value < 0.01. **indicates p-value < 0.001. ***indicates p-value < 0.0001; p-value < 0.05 were in bold font.

*Note*: Numbers in parentheses are 95% confidence intervals for estimated odds ratios.

^a ^The sample size reduced to 746 from 780 because the missing values of some factors used in the adjustment.

^b ^Adjusting for age, sex, race, residential areas, marital status, parental status, work status, income, and education, obesity, number of health problems, reported duration of illness, and medication use of OTC pain relieves

## Discussion

Forty percent of all subjects in our population-based study of fatiguing illnesses reported barriers to healthcare utilization. Only 14% of non-fatigued subjects reported a barrier, while 55% of those with CFS and 43% of those classified as ISF reported at least one barrier.

### Socio-demographic Trends and Barriers to Healthcare Utilization

Age, race, place of residence, marital status, income, and education were not significant factors in barriers to healthcare utilization. Women were more likely than men to report barriers to healthcare utilization regardless of women's higher frequencies of healthcare utilization in terms of general health and on several somatic symptoms: fatigue, sleep, and pain. A noteworthy finding as other studies shows that CFS is more prevalent in women than men. The higher barriers in seeking healthcare within women may be explained by the severity of their symptoms and their recognition of healthcare. Race and ethnicity alone also do not appear as significant barriers in seeking healthcare for fatiguing illnesses, a finding that differs from other chronic illnesses such as depression [9].

High-deductible health plans can be a good fit for relatively healthy people, but such health plans may also lead to people foregoing healthcare because they cannot afford the deductibles for unforeseen health conditions or illness. The uninsured impact is much bigger in people with fatiguing illness and concurrent psychiatric conditions, and may lead this group to forego healthcare frequently. However, foregoing healthcare when needed may lead to more serious and more expensive health problems in the future, and prove more costly to the individual in missing work, in reduced family responsibility, and health-related quality of life.

### Impact of Health History and Status on Healthcare Utilization

Six percent of the sample reported being diagnosed with CFS by healthcare professionals in the past. Nevertheless, 13 of 50 (26%) subjects with CFS history did not fulfill the CDC published diagnostic research criteria for CFS at the time of the clinical assessment in this study. The study showed that in seeking healthcare consultation, respondents with the history of CFS diagnosis were not significantly statistically different from those without the history of CFS diagnosis. Yet, respondents who fulfilled CDC published diagnostic research criteria for CFS at clinical assessment were more likely than non-fatigued respondents not to pursue needed healthcare consultation during the preceding year. These data lend support to previous research that found less than 20% of persons with CFS have been diagnosed [6], as in this sample those meeting CFS research criteria were less likely to seek healthcare compared to the control group. This situation presents a challenge and juxtaposition as persons with probable CFS are being under-diagnosed, and yet this population appears not to seek healthcare when needed or postpones seeking healthcare.

Over half of CFS subjects reported delayed help-seeking behavior. Although there was no statistically significant association between CFS history and healthcare under-utilization, regardless of the current CFS status, respondents with CFS history had higher utilization of healthcare consultation in symptoms associated with CFS – fatigue, sleep, cognitive dysfunction, and pain – than those who have not been diagnosed with CFS in the past. This suggests that higher healthcare utilization for CFS associated symptoms may be occurring for treatment and management issues even after a CFS diagnosis was given.

Subjects previously diagnosed as CFS by their physician had significantly worse physical health status (lower PCS score) than those without a formal diagnosis of CFS in the past. However, the previously diagnosed CFS subjects had significantly better mental health status (higher MCS score) compared to those without a previous diagnosis. This paradox suggests that receiving a CFS diagnosis may encourage seeking professional medical help and may also alleviate potential mental anxiety and stress about the origins of their illness. However, the cross-sectional data do not allow us to examine their health-related quality of life (measured by PCS and MCS) before or after receipt of the diagnosis of CFS by a physician, what treatment they received, and what information they received to help them for self-management of the illness. Further studies to explore this association are needed. Finally, co-existing medical or psychiatric conditions and obesity increased the chance of not pursuing healthcare consultation when needed. This result may be a confounder with socio-demographic factors such as uninsured, underinsured, and responsibilities of child care. The uninsured were more likely not to have sought healthcare services than were those who reported two or more forms of healthcare coverage.

### Why Do Barriers to Healthcare Utilization Occur?

The study qualitatively examined types of healthcare utilization barriers and found seven reoccurring barriers: attitudes, self-diagnosis, finance, time constraints, healthcare coverage, fear, and lack of trust and confidence in health professionals. Each of these types was listed as to reasons why subjects did not seek healthcare when needed. Results show that barriers as a whole were a significant issue for persons classified as CFS or "CFS but an exclusionary diagnosis". While not significant, a trend in barrier status is evident with non-fatigued subjects reporting the least barriers compared to those with some type of physical or mental health problems. When barriers were further classified into three domains – accessibility, KAB, and healthcare system – analyses indicate that for the CFS group, accessibility and healthcare system were the greatest barriers in seeking healthcare.

Lack of healthcare coverage is a known barrier to healthcare utilization and this study now supports this result in a population of fatiguing illnesses. Fifty-three percent (60) of uninsured study subjects reported barriers to healthcare utilization compared to 38% (255) of insured subjects (p-value < 0.01). Furthermore, 10% of study subjects reported foregoing healthcare because of financial barriers, which was nearly two-fold as that in the 2005 National Health Interview Survey (5%) [19].

Mistrust of healthcare professionals was significant among subjects classified with CFS. CFS subjects were less likely to seek healthcare and reported more barriers to healthcare utilization than other study groups. Mistrust stems from lack of confidence not only in healthcare professionals making a diagnosis but also in the structural barriers in the healthcare system such as the referral systems or perceived perceptions around a CFS diagnosis. As a consequence, subjects in the "healthcare system" domain were more likely to report making self-diagnosis and treating themselves with OTC medications further delaying seeking healthcare.

### Strengths and Limitations

The study's strengths include a rigorous study design with a random sample from a cross-sectional population-based study in fatiguing illness, which allows for a more generalizable inference of what can be achieved by identifying barriers to healthcare utilization and ways to eliminate or reduce barriers. This study employed both quantitative and qualitative research methods. Quantitative methods enabled the calculations of the prevalence of barriers to healthcare utilization, while qualitative methods allowed for in-depth analyses as to why subjects delayed health seeking behaviors.

One limitation of this study is that the sample was screened via random-digit-dial, computer-assisted phone screening in English and therefore excluded Spanish or other non-English speaking individuals. Other studies have shown that Hispanics or Latinos have at least as high prevalence of CFS as whites and yet they may not be represented in this analysis. The use of medications was documented for medications used in the two weeks prior to the clinic visit and makes it difficult to correlate to healthcare utilization in the preceding year. However, it is important to examine the association since the use of OTC pain relievers is the primary method in which study subjects self-treated pain symptoms. Another limitation is the possibility of recall bias on self-reported healthcare utilization data. This may be difficult to remedy and complex because recall bias may have affected the reporting of symptom occurrence, healthcare utilization and healthcare foregoing. These should be further explored in other studies.

We acknowledge that some of the findings from the current study may be attributed to socio-political and cultural contexts in the United States and those healthcare systems differ by country. In a cross-national study on perceived barriers to mental health, a cross-national effect was found in structural barriers and financial barriers; many more U.S. respondents (especially those with low incomes) reporting financial barriers than respondents in Ontario, Canada or the Netherlands [20]. However, in those countries, healthcare access and insurance differ greatly from the United States and these issues may act as confounders.

## Conclusion

This study shows that barriers to healthcare utilization pose a significant problem in persons with fatiguing illnesses. Health status, accessibility, and the healthcare system were barriers with the biggest impact on utilization. Results from the text analysis suggest that subjects may forgo needed medical care because they tend to self-diagnose their symptoms such as muscle and joint pains, headache, sleeping problems, etc. As a consequence of self-diagnosis, they self-treated their symptoms with over-the-counter medications. This approach may lead to more serious health problems later. Considering all of these, we suggest a two-fold model approach for future interventions: a symptom-targeted model and an interactive model. The symptom-targeted model would focus on symptoms associated with fatigue, such as fatigue, sleep, and pain, and encourage people experiencing these symptoms to seek healthcare promptly. Simultaneously it would recommend to healthcare professionals that patients seeking healthcare with these symptoms may need continued monitoring for potential CFS, chronic fatigue or other chronic conditions.

The interactive model would target patients and healthcare professionals with the goal of improving interactions between these two groups in terms of fatiguing illness. This model would emphasize the accurate diagnosis of underlying diseases thereby decreasing misdiagnosis and increasing CFS diagnosis in order to provide targeted therapy. Furthermore, it would encourage continued dialog between patients and healthcare professionals in terms of illness of management. These goals will be accomplished through education efforts aimed at health care practitioners enabling them to recognize the criteria for CFS diagnosis and management. Intervention for CFS should also address perceived stigma and trust for persons seeking healthcare in the area of CFS. Examples include reducing health professionals' barriers by increasing knowledge about CFS, increasing diagnostic self-efficacy and skills, and facilitating understanding of the patient's needs to reduce mistrust. This proposed model would also focus on improving individual perceptions of seeking healthcare consultation among persons with fatiguing illnesses with the goal to decrease unconstructive attitudes and beliefs that may act as barriers.

## Competing interests

The authors declare that they have no competing interests.

## Authors' contributions

JSL contributed to the conception, had primary responsibility for qualitative and quantitative analysis, interpretation of the data, and drafted the manuscript. DJB contributed to intellectual input to data interpretation and the emerged text theme categories, and critically revising the manuscript. RB collaborated in preparing the medication use of pain relievers, interpretation of the data, and critically revising the manuscript. JJ collaborated with others in designing the Healthcare Utilization questionnaire for fatiguing illness, and critically revising the manuscript. WCR was Principal Investigator of the study, collaborated with others in designing the study, writing the protocol, supervising field work, interpretation of the data, and revising the manuscript. All authors have read and approved the final manuscript.

## Pre-publication history

The pre-publication history for this paper can be accessed here:


